# Retinal Imaging Findings in Inherited Retinal Diseases

**DOI:** 10.3390/jcm13072079

**Published:** 2024-04-03

**Authors:** Giulia Corradetti, Aditya Verma, Jasaman Tojjar, Louay Almidani, Deniz Oncel, Mehdi Emamverdi, Alec Bradley, Sophiana Lindenberg, Muneeswar Gupta Nittala, SriniVas R. Sadda

**Affiliations:** 1 Doheny Eye Institute, Pasadena, CA 91103, USAjtojjar@doheny.org (J.T.); lalmidani@doheny.org (L.A.);; 2 Department of Ophthalmology, David Geffen School of Medicine at UCLA, Los Angeles, CA 90095, USA; 3 Department of Ophthalmology and Visual Sciences, University of Louisville, Louisville, KY 40202, USA; 4 Wilmer Eye Institute, Johns Hopkins University School of Medicine, Baltimore, MD 21205, USA; 5 Stritch School of Medicine, Loyola University Chicago, Chicago, IL 60153, USA

**Keywords:** adaptive optics, fundus autofluorescence, inherited retinal diseases, optical coherence tomography, precision medicine, retinal imaging

## Abstract

Inherited retinal diseases (IRDs) represent one of the major causes of progressive and irreversible vision loss in the working-age population. Over the last few decades, advances in retinal imaging have allowed for an improvement in the phenotypic characterization of this group of diseases and have facilitated phenotype-to-genotype correlation studies. As a result, the number of clinical trials targeting IRDs has steadily increased, and commensurate to this, the need for novel reproducible outcome measures and endpoints has grown. This review aims to summarize and describe the clinical presentation, characteristic imaging findings, and imaging endpoint measures that are being used in clinical research on IRDs. For the purpose of this review, IRDs have been divided into four categories: (1) panretinal pigmentary retinopathies affecting rods or cones; (2) macular dystrophies; (3) stationary conditions; (4) hereditary vitreoretinopathies.

## 1. Introduction

Inherited retinal diseases (IRDs) represent a heterogeneous group of genetic disorders and are reported to be the main causes of legal blindness in children and working-age individuals in many developed countries [1,2,3].

So far, more than 300 genes and loci have been associated with IRDs (RetNet, Retinal Information Network, https://sph.uth.edu/retnet, accessed on 1 November 2023). In addition, IRDs are characterized by a wide range of clinical and phenotypic presentations, and their heterogeneity constitutes a challenge in terms of diagnosis and the development of novel therapeutics [4,5].

Advances in gene therapy and retinal imaging have driven remarkable progress in the design of human clinical trials and clinical testing of novel therapeutics for IRDs [6,7,8,9]. This includes the utilization of small molecules, DNA- and RNA-based therapies, microelectrode arrays, and cellular transplantation [10,11,12,13]. As a result, the growth of clinical trials in IRDs and research in this field has exponentially increased over the last decade [14].

However, to date, there is a lack of consensus on the optimal outcome measures and surrogate endpoints for use in clinical trials [15,16]. The Monaciano Consortium, a group of worldwide experts on IRDs, was established to address the challenges and the existing gaps in diagnostics, natural history, the development of intervention clinical trials, and the standardization of novel outcome measures [14].

Changes in best corrected visual acuity (BCVA), visual field sensitivity, and retinal sensitivity measured by full-field stimulus testing (FST) are all considered to be efficacy endpoints that may be acceptable by the FDA, and are thus commonly used in IRD clinical trials, whereas the quantification of photoreceptor loss via optical coherence tomography (OCT) and the measurement of hypo- or hyper-autofluorescent lesions via fundus autofluorescence (FAF) are considered surrogate endpoints [14,17]. The identification of standardized, precise, and reproducible surrogate endpoints is important to achieve a better understanding of the natural history of this heterogeneous group of diseases and to improve the assessment of therapeutic efficacy. Consequently, there has been a growing interest in leveraging retinal imaging to uncover imaging endpoints, which could possibly be investigated as surrogate outcome measures in clinical trials. High-resolution, high-definition, and high-speed retinal imaging have revolutionized diagnosis and management strategies in ophthalmic diseases. These imaging advances have proved pivotal in the field of inherited retinal disease research.

The aim of this narrative review is to describe the advances in retinal imaging for patients with inherited retinal diseases, their characteristic imaging findings, and the imaging endpoint measures used in clinical research to serve as a guide for clinicians and trainees working in a clinical setting. The clinical relevance is to describe and highlight the imaging features we can uncover with a given imaging modality. Additionally, we have summarized the main imaging technologies and imaging outcome measures in clinical trials in the space of IRD. We believe that summarizing the main imaging modalities useful for each subgroup of IRDs and the related imaging findings is important for selecting the most informative diagnostics and interpreting them correctly.

In this narrative review, we have performed a broad literature PubMed search initially including the most relevant studies using the following terms: “retinal imaging OR inherited retinal diseases”, “optical coherence tomography OR inherited retinal diseases”, “fundus autofluorescence OR inherited retinal diseases”, “optical coherence tomography angiography OR inherited retinal diseases”, “clinical trials OR inherited retinal diseases”, “adaptive optics OR inherited retinal diseases”. Additionally, we searched for the terms “optical coherence tomography”, “fundus autofluorescence”, “adaptive optics”, “microperimetry”, “optical coherence tomography angiography”, and “clinical trials” in association (OR/AND) with each IRD described in the manuscript. Finally, we supplemented the search in some cases by adding references from relevant articles. All articles considered and cited were written in English.

For the purpose of this review, we will divide IRDs into four categories [18]: (1) panretinal pigmentary retinopathies primarily affecting rods or cones; (2) macular dystrophies; (3) stationary conditions; (4) hereditary vitreoretinopathies.

## 2. Panretinal Pigmentary Retinopathies

### 2.1. Cone Dystrophy and Cone–Rod Dystrophies (COD/CORD)

COD and CORD are a clinically and genetically heterogeneous group of IRDs, characterized by the primary degeneration of the cone photoreceptors, which may progress to the rods [19]. These diseases are classified as *stationary* (cone dysfunction syndromes), which usually have an early onset and purely involve the cone photoreceptors, and *progressive*, which are characterized by a later onset, and involve cone and rod photoreceptors. The cone dysfunction syndromes will be discussed in the “Stationary” Section 4. Retinal imaging, in particular FAF, represents a useful imaging technique to diagnose and monitor the progression of COD/CORD due to its ability to detect the concentration of lipofuscin and other fluorophores, signaling the pace at which the photoreceptor’s outer segments are metabolized by the retinal pigment epithelium (RPE) cells [20].

Adaptive optics (AO) has emerged as a useful imaging technology in recent case-based studies for imaging eyes with COD [21] and CORD [22,23,24]. Investigators have attempted to analyze the spatial orientation and topographical changes seen in photoreceptor cells in these conditions, with genotypic correlation in a few patients. For example, reduced cone density has been described in cases with *RP1L1* and *KCNV2* gene variants, while in a case with the *POC1B* gene variant, cones sparsely distributed around the fovea, but absent in other regions, have been documented [21,25,26]. Similarly, in cone–rod dystrophy, increased cone spacing (*RPGR*40, *CDHR1*43, *CEP250*90, and peripherin/RDS41 gene variants), sparsely observed bright cone photoreceptors in the ellipsoid zone (EZ), and enlarged photoreceptor inner segments surrounded by smaller-diameter photoreceptors (presumably rods) in areas of EZ disruption have been reported [24,27,28,29,30,31]. In fact, a cone spacing 0.7 times greater than the control group indicating a decreased cone density, 1 degree from the foveal center, has been suggested in eyes with CORD, implicating AO as a better imaging tool than OCT for monitoring early signs of progression in CORD. Further advantages of AO include higher sensitivity for detecting these changes and within shorter time intervals; thus, early progression can be monitored with great precision [32]. We will describe the imaging features of COD and CORD based on the following genotypes: *GUCA1A*, *GUCY2D*, *ABCA4*, *PRPH2*, and *RPGR.*

#### 2.1.1. GUCA1A-Associated COD/CORD

GUCA1A-associated COD/CORD (GUCA1A, OMIM #600364: autosomal dominant) is a variant causing late-onset COD and CORD and is characterized by heterogeneous macular RPE changes. FAF shows hyper-autofluorescence within the macular center, with a hyper-autofluorescent perifoveal ring, while atrophic areas are represented by the decreased autofluorescent signal [33,34].

#### 2.1.2. GUCY2D-Associated COD/CORD

GUCY2D-associated COD/CORD (GUCY2D, OMIM #600179: autosomal dominant) is characterized by early onset, and in color fundus photography, this variant shows non-specific RPE granularity and mottling within the macula during the early stages. The condition may progress to a bull’s eye maculopathy, and the end stage is characterized by atrophy (geographic atrophy) and peripheral chorioretinal atrophy with bone spicule pigmentation [35]. FAF shows increased foveal autofluorescence, with reduced autofluorescence in atrophic areas. During the more advanced stages, FAF shows a central hypo-autofluorescence corresponding with the atrophy. On OCT B-scans, a progressive loss of the EZ in the fovea, characterized by gradual thinning and atrophy of the adjacent retinal tissue, corresponds well to the fundoscopic picture [35,36].

#### 2.1.3. PRPH2-Associated CORD

PRPH2-associated CORD (PHRPH2, OMIM #179605: autosomal dominant) imaging findings can vary from a bull’s eye maculopathy to macular atrophy in fundoscopy. A typical “speckled” macular appearance can be appreciated in FAF in the majority of cases. OCT imaging is characterized by EZ attenuation and outer nuclear layer (ONL) atrophic cysts with foveal sparing [37,38].

#### 2.1.4. ABCA4-Associated COD/CORD

ABCA4-associated COD/CORD (ABCA4, OMIM #601691: autosomal recessive) is a phenotype characterized by mild changes in the fundus until advanced stages. A ring of increased autofluorescence surrounding a central zone of macular hypo-autofluorescence is a typical finding in FAF imaging, with sparing of the peripapillary area [39,40].

#### 2.1.5. RPGR-Associated COD/CORD

RPGR-associated COD/CORD (RPGR, OMIM #312610: X-linked recessive) phenotype is characterized by RPE atrophy in COD, while CORD cases mainly show peripheral involvement; however, overall, the fundus characteristics may vary. FAF shows a hyper-autofluorescent ring located perifoveally. These patients tend to show a progressive phenotype in most cases [41].

### 2.2. Rod–Cone Dystrophy (RCD) and Associated Diseases

RCD represents a heterogeneous group of IRDs, both genetically and clinically [42]. We will discuss the imaging findings of retinitis pigmentosa (RP), its variants, and associated disorders, including Leber congenital amaurosis and enhanced S-cone syndrome.

#### 2.2.1. Retinitis Pigmentosa (RP)

**Figure 1:**
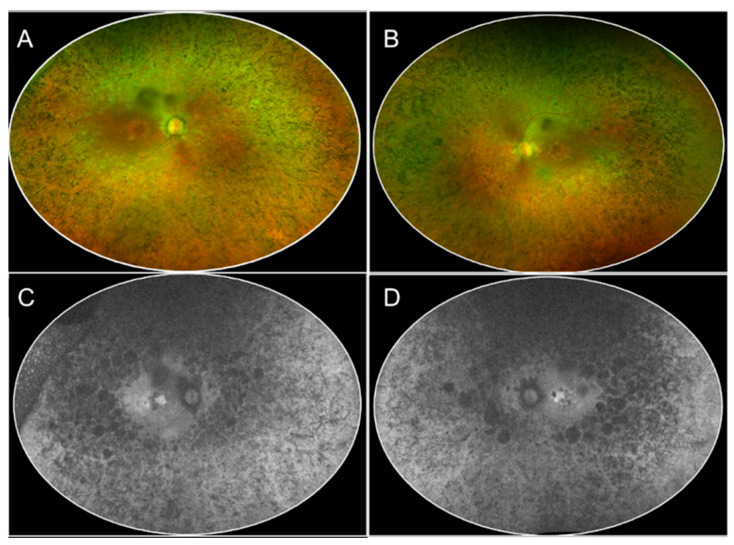
The multimodal imaging collage shows the color fundus photography (CFP, panel (**A**,**B**)) and fundus autofluorescence (FAF, panel (**C**,**D**)) phenotype in a 50-year-old patient affected by retinitis pigmentosa (RP). The color fundus photography highlights the presence of bone spicule pigmentation that is distributed 360° in the midperiphery with macular sparing, vascular attenuation, and optic disc pallor, characteristic of the classic presentation of RP (Panel (**A**,**B**)). In FAF imaging, a diffuse patching reduction of autofluorescence is noted with rounded patches of atrophy closer to the vascular arcades, indicating a loss of autofluorescence (Panel (**C**,**D**)).

**Figure 2:**
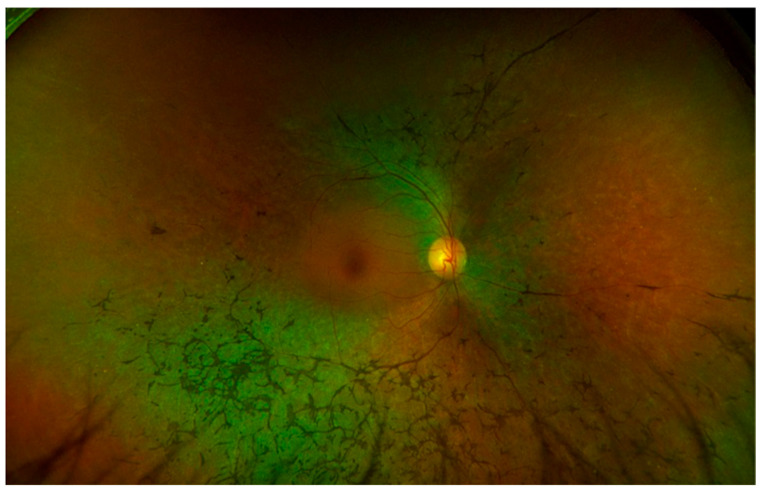
Ultra-widefield color image of a case of sectoral Retinitis Pigmentosa, showing bone spicule pigmentation inferotemporal but not superiorly.

The term RP is used to describe a heterogenous group of IRDs for patterns of inheritance and it is considered the most prevalent phenotype of IRD [43]. The disease is associated with progressive dysfunction of the rods, which extends to the cone photoreceptors and RPE, with vascular attenuation and choroidal atrophy. Patients with RP complain of nyctalopia and loss of peripheral vision with tubular visual fields. Upon fundus examination, eyes with RPE show waxy pallor of the optic nerve, narrowing of the retinal arterioles, mottling of the RPE with the presence of pigment clumping and typical bone spicule pigmentation, which is believed to represent the migration of pigment into the interstitial and perivascular spaces of the retina (Figure 1) [44]. Advances in retinal imaging have highlighted the importance of multimodal imaging to achieve more precise quantitative measurements and to uncover new imaging endpoints to improve the management and follow-up of the disease over time. OCT imaging studies have highlighted that the attenuation of the foveal EZ can predict the state of rod degeneration, as well as visual acuity [45]. In addition, EZ changes as measured via en face OCT have also been used as a marker for the progression of the disease in clinical trials [46]. EZ width as a measure of the integrity of the photoreceptors and evaluated via OCT B-scans was used as an outcome imaging measure in a phase I trial (NCT03063021) assessing the safety and tolerability of oral N-acetylcysteine (NAC) in patients with RP, showing no significant decline in the EZ width in any of the cohorts [47]. Intraretinal hyper-reflective foci are considered negative prognostic factors for visual preservation in RP [48]. The status of the choroid in RP has also been investigated. RP eyes with cystoid macular edema (CME) were found to manifest a thicker choroid [49]. In FAF imaging, eyes with RP frequently show a typical ring of increased autofluorescence within the parafoveal area, which is believed to represent the boundary between the impaired and preserved retina [50]. Ultra-widefield (UWF) FAF is particularly important for assessing the presence of abnormal FAF patterns, decreased autofluorescence (DAF), and the extent of autofluorescence in RP as main outcome measures [42,51,52]. OCTA allows for the quantification of microvascular changes within the retina. In cases of RP, an enlargement of the FAZ has been reported, as well as reduced perfusion density in both the superficial and deep capillary plexus [53,54]. In addition, impairment at the level of the choroidal microcirculation has been associated with EZ attenuation and worse visual acuity in RP [55]. Several studies have shown a decrease in cone density in AO imaging [24,42,56]. Sectoral RP, a variant of RP, is characterized by a disease pattern similar to the classic form but confined to one or two quadrants, and it is associated with a better prognosis (Figure 2) [57].

#### 2.2.2. RPGR-Associated RP

The most severe form of RP in males is associated with a mutation on the RPGR gene, and represents the most common cause of X-linked (XL) RP [58] (RPGR, OMIM #312610: X-linked). A typical imaging finding of this form of RP is the presence in FAF of a perifoveal ring of hyper-autofluorescence [42].

#### 2.2.3. RP2-Associated RP

RP2 represents a gene causing another form of XL RP (RP2, OMIM #300757y: X-linked) that affects males with early-onset severe retinal degeneration, early macular involvement, and consequently severe visual function loss due to the loss of foveal photoreceptors. OCT imaging is helpful to identify and quantify the loss of photoreceptors in terms of EZ area loss, decreased ONL thickness, and atrophy of the RPE. FAF will show progressive atrophy depending on the stage of the disease [42].

#### 2.2.4. USH2A-Associated RP

USH2A variants (USH2A, OMIM #608400: autosomal recessive) can cause a form of RP associated with neurosensory hearing loss (Usher syndrome) or isolated RP. Imaging findings in this variant are similar to the classic form of RP, described in the previous section. Recently, Duncan et al. have shown that the degree of peripheral degeneration can predict central vision loss. Therefore, AO imaging has been described as a valuable imaging technique to monitor and characterize the progressive degeneration of the cones in advanced disease [59].

#### 2.2.5. RLBP1-Associated RP

The RLBP1 gene causes an autosomal recessive phenotype of RP (RLBP1, OMIM #180090: autosomal recessive). It is common to identify retinal white dots in color fundus photography, as well as progression to atrophy during the late stages. FAF typically shows globally reduced autofluorescence [60].

#### 2.2.6. NR2E3-Associated RP

Mutations on the NR2E3 gene have been described with an autosomal dominant form of RP (NR2E3, OMIM #604485: autosomal dominant) showing a phenotype similar to classic RP with initial impairment of the rods and subsequent involvement of the cones, and the presence in fundus photography of bone spiculae in the midperiphery [61].

### 2.3. Leber Congenital Amaurosis (LCA)

LCA comprises a group of early-onset childhood IRDs clinically characterized by early vision loss, nystagmus, and severe generalized retinal degeneration. This group of diseases has shown genetic and phenotype heterogeneity. We will discuss the LCA variants associated with *RPE65*, *CRB1*, *GUCY2D*, *CEP290*, and *RDH12.*

#### 2.3.1. RPE65-Associated LCA

RPE65-associated LCA represents the first IRD that has been explored for gene therapy (RPE65, OMIM #180069: autosomal recessive), and an FDA/EMA-approved gene therapy is available for this disease. Fundus photography shows generalized RPE degenerative changes, pigment epithelial hyperplasia, and patchy atrophy, which correspond in FAF to large regions of reduced or absent autofluorescence. Retinal structural changes have been described in OCT studies. To assess retinal structural changes, OCT imaging was employed in the phase 1 trial assessing the treatment of RPE65-associated LCA by the ocular subretinal injection of adeno-associated virus gene vector [62]. Specifically, foveal thickness was used as an outcome measure before and after treatment. Full-field light sensitivity threshold testing (FST) was performed to measure the lowest illumination perceived in both phase 1 and phase 3 clinical trials to assess the function of the rod photoreceptors, showing a gain in FST following treatment [63].

#### 2.3.2. CRB1-Associated LCA

**Figure 3:**
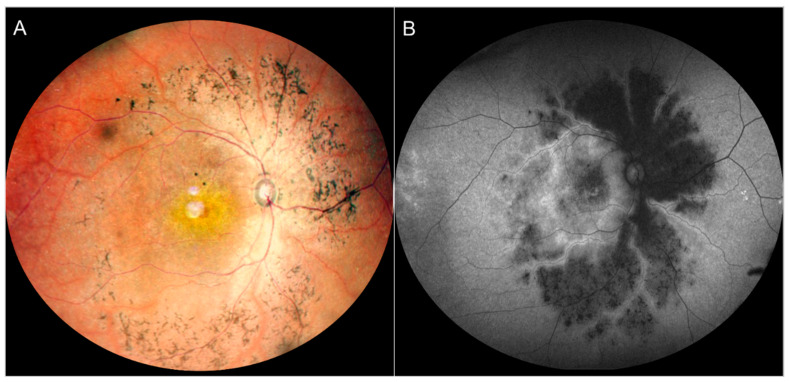
A case of a 27-year-old female with preserved para-arteriolar retinal pigment epithelium (PPRPE) presentation related to a CRB1 mutation. The sparing of the para-arteriolar retinal tissue is evident in the color fundus photograph (panel (**A**)) by the presence of the pigmentation surrounding the veins but not the arteries, which corresponds to a typical autofluorescence pattern with para-arteriolar sparing (panel (**B**)).

Mutations on the CRB1 gene (CRB1, OMIM #604210: autosomal recessive) have been associated with a form of LCA characterized by the para-arteriolar preservation of the RPE (PPRPE) [64]. FAF imaging highlights the characteristic para-arteriolar sparing pattern of this variant, which is considered a less severe form of the disease with slower progression [64]. OCT studies have described the association between the CRB1 mutation and an abnormal retinal lamination (Figure 3) [65,66].

#### 2.3.3. GUCY2D-Associated LCA

GUCY2D-associated LCA (GUCY2D, OMIM #600179: autosomal recessive) is characterized by a relatively normal fundus with a preserved outer retina as documented by OCT imaging during the early stages [67]. The integrity of the outer retina has been evaluated in terms of EZ, RPE integrity, and ONL thickness [67,68].

#### 2.3.4. CEP290-Associated LCA

Mutations on the gene CEP290 (CEP290, OMIM #610142: autosomal recessive) cause CEP290-associated LCA characterized by the early and severe dysfunction of cone photoreceptors, and are the gene defects that represent the majority of LCA cases with severe blindness [69]. Despite early functional impairment of the cones, OCT has demonstrated the retina to be relatively structurally intact during the early stages [70]. Phase 1 and phase 2 clinical trials are ongoing, and a key outcome measure involves the measurement of light sensitivity [71].

#### 2.3.5. RDH12-Associated LCA

RDH12 mutations (RDH12, OMIM #608830: autosomal recessive or autosomal dominant) are responsible for a form of LCA characterized by a severe and progressive rod–cone dystrophy with macular atrophy. Fundus photography shows attenuation of the retinal arterioles, the presence of intraretinal spicule pigmentation, and outer retinal atrophy [72]. OCT findings are characterized by outer retina atrophy showing an excavated appearance in the most advanced cases. However, the extent of the atrophy is variable from macular atrophy to generalized atrophy involving the posterior pole and the periphery [73].

### 2.4. Enhanced S-Cone Syndrome (ESCS, or Goldman-Favre Syndrome)

ESCS (NR2E3, OMIM #268100: autosomal recessive) is a slowly progressive IRD characterized by increased sensitivity to blue light due to an overexpression of the S (short wavelength, blue) subtype of cone photoreceptors. The predominance of S cones is associated with an alteration in the normal ratio of S to L (long wavelength, red) and M (middle wavelength, green) cone subtypes, which occurs during early development [61]. Color fundus photography shows symmetric findings including typical nummular pigmentation, characterized by deep round patches of RPE hyper-pigmentation distributed in the midperiphery, just outside the vascular arcades, and associated with RPE atrophy, corresponding in FAF to a loss of autofluorescence [74]. In OCT, nummular pigmentary deposits and associated atrophy are manifested as EZ and RPE loss. These findings, despite being frequent in ESCS, are not pathognomonic, and they are detected in other IRDs such as CRB1-associated LCA [75]. In some cases, fundus color photography shows white-yellowish dots forming a ring in the midperiphery, usually sparing the macula, corresponding to hyper-autofluorescence in FAF. Histological studies have highlighted that the autofluorescent signal from the white-yellowish dot pattern is derived from macrophages, topographically corresponding to dysplastic photoreceptors [76]. Torpedo-like lesions have also been described in association with ESCS [74,77,78].

### 2.5. Choroideremia (CHM)

**Figure 4:**
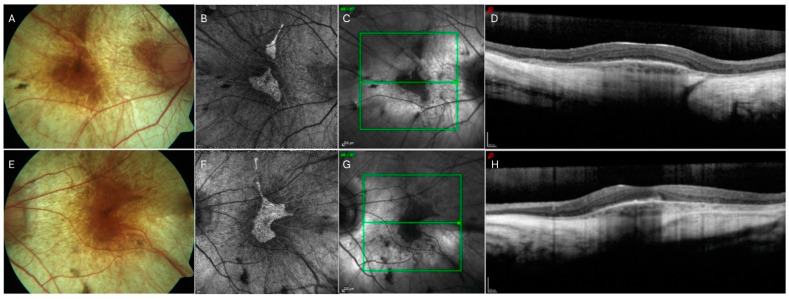
Collage illustrating multimodal imaging in a case of choroideremia (CHM). The standard color photograph (panel (**A**,**E**)) shows diffuse whitish color due to diffuse choroidal and retinal atrophy surrounding the posterior pole, allowing the color of the sclera to be seen. Of note, the color photographs show a typical stellate preservation of the posterior pole, which is characteristic of the disease, and also evident in the corresponding in the fundus autofluorescence (FAF, panel (**B**,**F**)), in which a well-defined area of hyper-autofluorescence with sharp margins is seen. The infrared images (panel (**C**,**G**)) match the color fundus photograph topography of the atrophy. The OCT B-scans highlight the relative central preservation, with severe thinning of the surrounding choroid (panel (**D**,**H**)).

Choroideremia (CHM) (REP1, OMIM #300390: X-linked recessive), an uncommon inherited retinal ailment arising due to mutations in the CHM (RPE1) gene, necessitates a diverse array of imaging indicators for both its diagnosis and treatment. It is a rare X-linked recessive disorder that results in the progressive degeneration of the RPE, photoreceptors, and choroid (Figure 4) [79]. OCT and FAF allow for reliable measurements of the area of preserved EZ and preserved RPE, respectively, which can be considered as reliable quantitative outcome metrics for the anatomic progression in CHM clinical trials [80]. Of note, while preserved EZ and RPE areas are highly correlated, the preserved EZ area appears significantly larger than the preserved RPE area in patients with CHM, suggesting that the RPE degenerates faster than the photoreceptor layer [80]. These findings are in agreement with other studies reporting that functional cones are observed beyond the border of areas of decreased autofluorescence, but not beyond the border of areas with EZ preservation [81,82]. OCTA studies have also shown that choriocapillaris preservation highly correlates with photoreceptor structural preservation in CHM [54]. However, the areas of choriocapillaris impairment appear less extensive than the areas of RPE loss, supporting the hypothesis that RPE damage in CHM precedes photoreceptor and choriocapillaris loss [83].

Cone spacing assessed via AO has suggested that cone structure is preserved extending to the edge of atrophy [81]. Functional studies using fundus-guided microperimetry have also shown that retinal sensitivity persists beyond the FAF and OCT border of RPE and EZ preservation [81]. AO-guided microperimetry studies have shown that retinal sensitivity is preserved within areas with contiguous cone mosaics, while dense scotomas are evident in atrophic areas [84]. Fundus-guided microperimetry has been performed in clinical trials evaluating treatments for CHM; however, given the advanced stage of the disease in these participants, the microperimetry data were generally found to be inconsistent [85,86,87]. Thus, to summarize, the main outcome measures to assess retinal structure adopted in clinical trials testing gene therapy in choroideremia are EZ preservation area and RPE preservation area evaluated by OCT and FAF, respectively [86,87,88,89].

### 2.6. Gyrate Atrophy

Gyrate atrophy (OAT, OMIM #613349: autosomal recessive) is a rare IRD characterized by progressive vision loss due to mutations in the ornithine aminotransferase (OAT) gene [90,91]. Gyrate atrophy is characterized by the development of patches of chorioretinal atrophy starting in the midperiphery of the retina during the first decade of life, and then extending anteriorly and posteriorly as the disease progresses [92]. In color fundus photography, the areas of atrophy in gyrate atrophy show typical scalloped margins, which correspond to areas of reduced autofluorescence in FAF [93]. It has been shown that microperimetry retinal sensitivity is detectable over areas of preserved RPE as measured by FAF [94]. These preserved RPE areas have also been shown in OCT to show preservation of the photoreceptors [94]. In addition, eyes with gyrate atrophy can show the presence of intraretinal cystoid spaces and foveoschisis in OCT imaging [94,95]. OCTA studies have reported an enlarged FAZ in gyrate atrophy, which seems to correlate with the detection of intraretinal cystoid spaces in OCT [96].

## 3. Macular Dystrophies

### 3.1. Stargardt Disease (STGD)/Fundus Flavimaculatus

**Figure 5:**
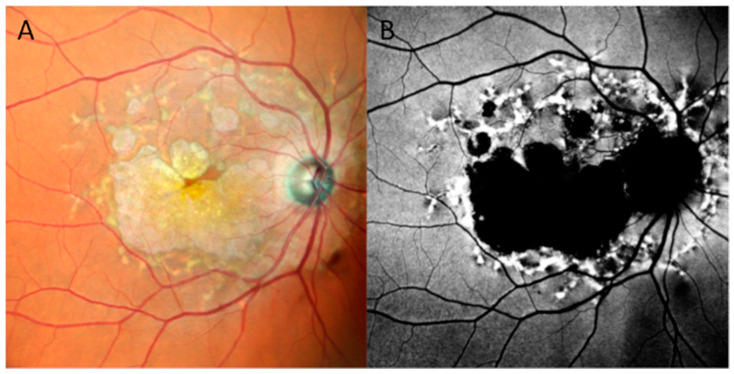
This collage illustrates multimodal imaging of a classic form of Stargardt with the typical beaten-metal appearance of atrophy surrounded by flecks in color fundus photography (panel (**A**)), and fundus autofluorescence (panel (**B**)).

**Figure 6:**
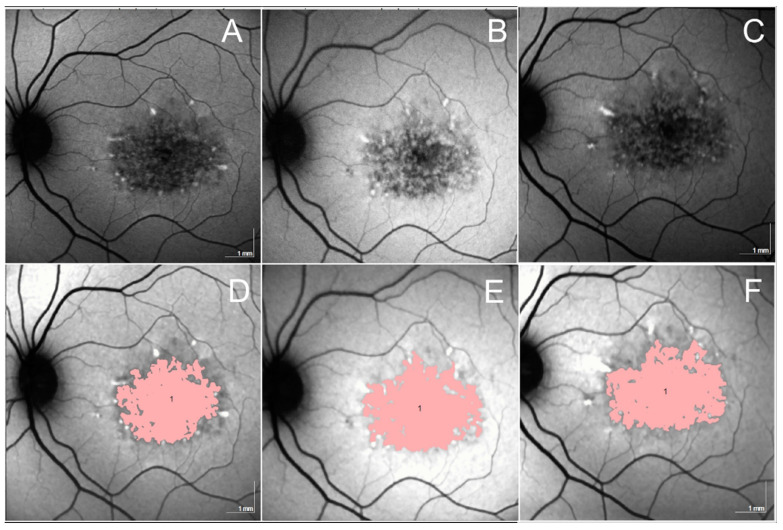
The panels illustrate examples of lesions of the total region of decreased autofluorescence (DAF) in an eye affected by Stargardt disease. Upper row, panels (**A**–**C**) illustrate the decreased autofluorescence pattern in FAF images at baseline, 12- and 24-month follow-up, respectively. Bottom row, panels (**D**–**F**) illustrate the total area of decreased autofluorescence at baseline, 12-, and 24-month follow-up. The lesion is characterized by a total DAF area measuring 5.99 mm^2^ at baseline (panel (**D**)), 6.86 mm^2^ at 12 months (panel (**E**)), and 7.05 mm^2^ at 24 months (panel (**F**)).

STGD (STGD1, ABCA4, OMIM #601691: autosomal recessive homozygous or heterozygous; SGDT4, PROM1, OMIM #604365: autosomal dominant heterozygous; STGD3, ELOVL4, OMIM #600110: autosomal dominant STGD-like macular dystrophy) is the most common form of macular dystrophy in young adults with onset typically in childhood or early adulthood. The late-onset/foveal-sparing STGD form is characterized by onset in later adulthood [97]. Patients present with reduced central vision and characteristic fundus flecks, which are lipofuscin deposits in RPE [98]. The clinical stages of the disease are based on the fundus appearance. Stage 1 is characterized by a beaten-metal appearance confined to the macula associated with a discontinuous ring of flecks around the fovea. Stage 2 is characterized by widespread flecks beyond temporal arcades and/or nasal to the disc. At this stage, we register an abnormal cone and rod response. Stage 3 is characterized by the re-absorption of flecks and widespread choriocapillaris impairment. Finally, stage 4 demonstrates further reabsorption of flecks, with extensive choriocapillaris atrophy associated with RPE atrophy [99]. The macular atrophy in eyes affected by STGD is clearly illustrated in FAF represented by an area of definite hypo-autofluorescence defined by sharp margins centered on the macula and surrounded by hyper-autofluorescent flecks (Figure 5). FAF imaging is considered the gold standard to monitor the growth of atrophy in STGD. The identification of areas of definite decreased autofluorescence and the total combined area of decreased autofluorescence as measured by FAF is considered the preferred structural endpoint in clinical trials (Figure 6) [100,101]. Of note, ultra-widefield FAF in ABCA4 STGD disease is useful to assess and define the clinical pattern, especially to identify cases with peripheral changes, which may have implications for staging, prognosis, and eventually management [102,103]. Optical coherence tomography (OCT) has proved to be useful to assess the presence and extension of the photoreceptors, RPE, and choriocapillaris atrophy, and its extent. In addition, the area of preservation or loss of the ellipsoid zone (EZ) loss has also been used for longitudinal monitoring of the progression of the disease [104,105]. In vivo cellular imaging using adaptive optics (AO) allows for the identification of photoreceptor spacing and cone density [106]. Deep-learning segmentation algorithms have also been developed to measure the thickness of various retinal layers in eyes with Stargardt disease in an automated fashion, and this may also prove to be useful for monitoring disease progression [107]. Additional deep-learning tools are under development to define early risk factors or biomarkers that could predict STGD disease progression and the development of flecks [108]. Given that the best corrected visual acuity (BCVA) is not well-suited to serve as a functional endpoint in STGD disease, microperimetry has been investigated in multiple studies. Microperimetry plays a central role, especially in the early stages of the disease, prior to obvious regions of atrophy becoming evident, or prior to vision decline [109,110]. Rates of mesopic and scotopic retinal sensitivity have also been reported, showing that the loss of scotopic macular function preceded and appeared to be faster than mesopic deterioration [111]. However, fixation parameters did not appear to be sensitive functional outcome measures in clinical trials, but could be used in select cases to achieve a better understanding of the functional state [112]. Finally, microperimetry retinal sensitivity deterioration appears to correlate well with decreased choriocapillaris flow deficits as measured by OCTA [113]. Several clinical trials have been conducted or are underway to assess potential treatments for Stargardt disease, and imaging biomarkers have been deployed as endpoints. For example, intravitreally delivered complement inhibitors are being tested as potential treatments in Stargardt disease, with a phase 2 trial of the C5 inhibitor avacincaptad pegol underway [114]. While early trials of the complement C5 inhibitor eculizumab were unsuccessful, it is hoped that the localized intravitreal delivery of avacincaptad pegol may confer greater efficacy [114]. The primary endpoint of the study is the mean rate of change of the area of ellipsoid zone defects as measured by OCT [115]. Natural history studies employing quantitative fundus autofluorescence have demonstrated symmetry between eyes and provided robust structural endpoints to track disease progression, particularly in childhood-onset Stargardt disease [116].

### 3.2. Bestrophinopathies

Bestrophinopathy represents a term to define a heterogenous group of IRDs characterized by mutations of the BEST1 gene encoding for bestrophin-1 [117]. Mutations of the BEST1 gene can manifest as at least five different variants: (1) Best’s disease (Best vitelliform macular dystrophy; BVMD); (2) autosomal recessive bestrophinopathy (ARB); (3) Adult-Onset Vitelliform Macular Dystrophy (AOVMD); (4) autosomal dominant vitreoretinochoriodopathy (ADVIRD); (5) retinitis pigmentosa (RP). Given these heterogeneous clinical phenotypes, it is important to recognize that bestrophinopathies can involve the macula and periphery. In this section, we will focus on the phenotypes involving the macula.

#### 3.2.1. Best’s Disease (BD)/Best Vitelliform Macular Dystrophy (BVMD)

**Figure 7:**
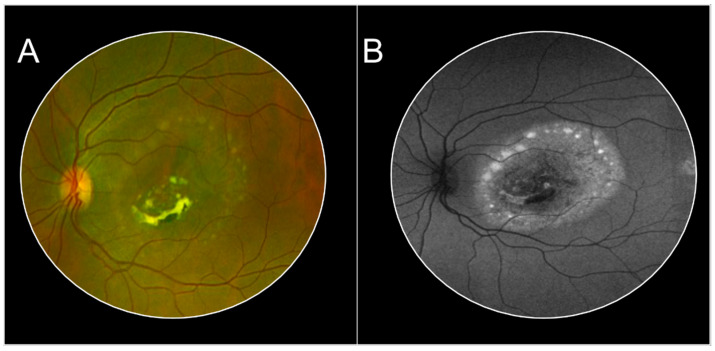
Case of a 23-year-old female with genetically confirmed Best’s disease. Color fundus photography shows the subretinal yellowish deposits in a scrambled egg appearance in the center surrounded by small round vitelliform lesions in a ring-like pattern (Panel (**A**)). This pattern is reproduced on the fundus autofluorescence (Panel (**B**)).

BVMD (BVMD, BEST1, OMIM #607854: autosomal dominant) represents the most common bestrophinopathy. The clinical presentation may vary based on the stage of the disease. The original classification of the disease, introduced by Gass, [118] was based on fundoscopy, and included five different stages: (1) *stage 1*, *subclinical*: absence of fundus abnormalities; (2) *stage 2*, *vitelliform*: presence of a subretinal macular lesion, half to two-disc diameters in size, filled by yellowish material resembling an egg yolk; (3) *stage 3*, *pseudohypopyon*: persistence of yellowish material in the inferior portion of the lesion; (4) *stage 4*, *vitelliruptive*: breakdown of the material, conferring a “scrambled egg” appearance to the macula; (5) *stage 5*, *atrophic*: macular lesion complicated by the development of chorioretinal atrophy, fibrosis, or macular neovascularization. Multimodal imaging is pivotal and varies with the stage of the disease (Figure 7) [119,120]. The clinical features of the various stages can be assessed via color fundus photography (CFP). Fundus autofluorescence is useful to assess the presence and distribution of the vitelliform material, which appears hyper-autofluorescent. In the early stages of the disease, the hyper-autofluorescence of the vitelliform material appears to be enhanced, whereas as the vitelliform material deposits towards the inferior portion of the lesion, and the hyper-autofluorescence of this inferior portion becomes more intense and creates a distinct pseudohypopyon level. During the late stages of the disease, a progressive loss of the hyper-autofluorescent signal can be detected [121]. The vitelliform stage appears on cross-sectional OCT imaging as a dome-shaped hyper-reflective lesion within the subretinal space. In contrast, the vitelliruptive stage appears in OCT as a regression of the subretinal hyper-reflective vitelliform material, the presence of hypo-reflective subretinal fluid, and the elongation of the photoreceptor outer segments. OCT imaging is also useful to depict the last stage of BVMD with fibrosis, atrophy, scarring, and/or macular neovascularization. Therefore, OCT B-scans can display and differentiate each of the stages of the BVMD, and are useful for the longitudinal monitoring of the disease [122,123,124,125]. Several studies have suggested that progressive enlargement of the area of EZ loss is associated with greater BCVA deterioration [126]. The extent of hyper-reflective foci as detected on OCT B-scans is highly correlated with the stage of BVMD, and they tend to increase as the disease progresses and are found in the greatest number in stage 4 [127]. Given that BVMD can be associated with MNV during stage 5, OCTA imaging is particularly important to detect the presence, extension, and features of the MNV lesion, as fluorescein angiography may be difficult to interpret in the face of staining of the vitelliform material with fluorescein dye [54,128]. In addition, the OCTA has been studied to assess the state of the choroid and choriocapillaris, and the inner retinal vascular layers in BVMD. Decreased perfusion density at the level of the deep capillary plexus (DCP) has been associated with a more rapid progression of the disease [129]. While the DCP has been found altered starting at stage 3 of the disease, choriocapillaris impairment is already present during the subclinical stage [130]. However, changes in the choriocapillaris impairment appear to become more severe with the development of atrophy [131]. In AO studies, the disruption of the EZ band identified by OCT imaging corresponds to decreased cone density, while the surrounding retinal tissue appears to be normal [132]. Functional studies using microperimetry have shown a correlation between retinal sensitivity and structural lesion appearance [133]. A trend toward a decreased retinal sensitivity has been observed as the vitelliform material is reabsorbed and subretinal fluid or atrophy develops. In addition, a strong correlation has been reported between the microperimetry retinal sensitivity and the outer nuclear layer thicknesses [134].

#### 3.2.2. Autosomal Recessive Bestrophinopathy (ARB)

ARB (BEST1, OMIM #607854: autosomal recessive) is characterized by the absence of the typical clinical appearance of BVMD [135]. In fundoscopy, these patients can reveal the presence of a central serous detachment associated with a fibrous subretinal central scar [136]. The distribution of the vitelliform lesions also differs from the BVMD form of the disease, as the vitelliform deposits tend to distribute proximal to the arcades. FAF is pivotal to assess the presence of the vitelliform lesions, and the hyper-autofluorescent signal appears to achieve a greater intensity for smaller lesions. OCT imaging highlights both the subretinal fluid associated with the serous detachment, as well as the presence of the vitelliform lesion, and macular neovascularization [136].

#### 3.2.3. Adult-Onset Vitelliform Macular Dystrophy (AOVMD)

AOVMD (BEST 1, OMIM #608161: autosomal dominant) represents a subtype of BVMD, which differs from the classical form by the late onset of the disease. Although AOVMD can be inherited and associated with BEST 1 or PRPH2, the majority of cases are idiopathic. AOVMD is characterized by minimal or more slowly progressive vision deterioration, and the clinical phenotype is similar to milder forms of BVMD. FAF and OCT facilitate the visualization of the vitelliform lesions and their location. Given the similarity between AOVMD and BVMD, inherited cases of AOVMD that are found to have a BEST1 mutation are often re-classified as BVMD [136].

#### 3.2.4. Pattern Dystrophy (PD)

PD (PRPH2, OMIM #179605: autosomal dominant) refers to a heterogeneous group of macular disorders of the RPE characterized by an abnormal accumulation of lipofuscin in and around the RPE, mostly within the macular region [137]. On fundoscopy, these deposits appear yellowish or brown, and can be associated with RPE mottling within the macular area. The lipofuscin accumulation can resemble different shapes and sizes, giving the fundus a characteristic speckled pattern, or reticular pattern. In FAF, these lesions appear hyper-autofluorescent, while they are described in OCT imaging as subretinal hyper-reflective material. OCT imaging allows not only for the visualization of these deposits, but also for the precise definition of the phenotype and the location of the material in PD [137]. The areas with RPE depigmentation are detected in FAF as being hypo-autofluorescent. OCT B-scans can show macular neovascularization or scarring in advanced stages. A form of AOVMD associated with PRPH2 mutation is considered a subtype of PD [136].

#### 3.2.5. Sorsby Fundus Dystrophy (SFD)

SFD (TIMP3, OMIM #188826: autosomal dominant) is a macular dystrophy characterized by central vision loss during the fourth or fifth decade of life [138,139]. At onset, the fundus shows sub-RPE drusen-like deposits that resemble reticular pseudodrusen, which usually spare the fovea. In OCT, these deposits appear similar to subretinal drusenoid deposits. During the advanced stages of the disease, the phenotype is complicated by atrophy and macular neovascularization [140].

#### 3.2.6. North Carolina Macular Dystrophy (NCMD)

NCMD (MCDR1, OMIM #616842: autosomal dominant) is a congenital, bilateral macular dystrophy with onset during the first decade of life. The fundus lesion appears to be bilateral and symmetric and characterized by drusen-like deposits (grade 1) that tend to become confluent (grade 2), and progress to RPE atrophy associated with choroidal excavation and a colobomatous-like appearance (grade 3). The end stage of the disease can also be complicated by macular neovascularization, fibrosis, and scarring [141,142]. OCT imaging reveals with high precision and resolution the abrupt termination of the photoreceptors and RPE bands, and the choroidal excavation in the colobomatous-like stage of the disease [141].

#### 3.2.7. Central Areolar Choroidal Dystrophy (CACD)

CACD (CACD1, GUCY2D, OMIM #600179: autosomal dominant; CACD2, PRPH2, OMIM 179605: autosomal dominant) presents with a well-demarcated area of atrophy of the RPE and choriocapillaris, usually centered in the macula [143,144,145]. FAF has proved to be an important imaging modality to identify the accumulation of lipofuscin as well as RPE hypo-pigmentation. The complete loss of all the outer retina layers is well identified and imaged by OCT B-scans. OCTA is useful in detecting the state of the choriocapillaris, and it has been described that eyes affected by CACD show patchy areas of choriocapillaris flow deficits not only in the area corresponding with the RPE atrophy but also in the surrounding area [145]. These phenomena seem to correlate with histological models, showing that the choriocapillaris damage is subsequent to the destruction of the RPE [146].

#### 3.2.8. Autosomal Dominant Occult Macular Dystrophy (OCMD)

OCMD (hereditary OCMD, RP1L1, OMIM #608581: autosomal dominant,) represents a rare form of inherited macular dystrophy, which affects primarily the foveal cones bilaterally and is associated with progressive visual acuity loss [147,148].

Patients affected by this condition may present with a normal fundus with a blunted foveal reflex. OCT imaging captures the attenuation or disruption of both the ellipsoid zone and interdigitation zone of the photoreceptors at the level of the fovea, suggesting a degeneration of the inner and outer segments of the photoreceptors [149]. In multiple reports, AO imaging measured decreased cone densities in eyes with hereditary OCMD, confirming that the observed cell signals were derived from cone photoreceptors [150]. However, a similar clinical presentation has been reported in non-hereditary OMD or hereditary OMD not associated with the RP1L1 gene mutation. Therefore, OCMD can be classified as hereditary autosomal dominant RP1L1-associated OCMD (or Miyake disease), hereditary OCMD associated with other gene abnormalities, and non-hereditary OCMD-like syndrome (progressive OCMD) [151].

#### 3.2.9. Doyne Honeycomb Retinal Dystrophy/Autosomal Dominant Drusen (ADD)

ADD (EFEMP1, OMIM #601548: autosomal dominant) is characterized by the presence of soft drusen of different sizes, distributed both within the macular and peripapillary areas, resembling a “honeycomb” pattern. Large round drusen are mainly located in the perimacular and peripapillary area, whereas smaller drusen showing a radial distribution are mostly located temporal to the macula [152]. FAF imaging highlights the hyper-autofluorescence of the drusen, while the hyper-pigmentation appears hypo-autofluorescent. OCT imaging allows for a more precise characterization of the drusen: large drusen show a dome shape with overlying attenuation of the RPE layer; small drusen appear as an irregular thickening of the RPE/Bruch membrane complex; and the RPE overlying the drusen appears relatively preserved. Cases of macular neovascularization as a late complication of the disease have been reported [152].

## 4. Stationary Conditions

### 4.1. Complete and Incomplete Congenital Stationary Night Blindness (CSNB)

CSNB is a clinically and genetically heterogeneous group of non-progressive IRDs, characterized by impaired night vision. CSNB is classified into two groups based on the presence of fundus abnormalities. The forms of CSNB with *normal fundus* appearance are subsequently classified into two groups: (1) the *Schubert–Bornschein CSNB type*; (2) the *Riggs CSNB type*. Clinically, the Schubert–Bornschein type presents with reduced visual acuity, myopia, and nystagmus, which are not present in the Riggs type. *Fundus albipunctatus* and *Oguchi disease* represent the two entities of CSNB with an *abnormal fundus* appearance [153]. OCT imaging is useful in the evaluation of fundus albipunctatus and Oguchi disease. OCT shows hyper-reflective deposits between the RPE and the outer nuclear layer in eyes affected by fundus albipunctatus. These deposits seem to correspond to the dot-like deposit visualized in fundoscopy. The fundus in Oguchi disease is characterized by a typical golden-yellow discoloration, which disappears after prolonged dark adaptation (Mizuo–Nakamura phenomenon) [154]. OCT imaging shows a shortening of the rod outer segments in the periphery, though the distance between the EZ and RPE in the macula is not decreased [155]. AO imaging has highlighted that the signal intensity after dark adaptation only changes within the rods, suggesting isolated impairment within the rods, and not the cones [156].

### 4.2. Congenital Achromatopsia (ACHM)

ACHM (ACHM3, CNGB3, OMIM #605080: autosomal recessive) is a bilateral, symmetric IRD, that is characterized by the absence of all three types of cone photoreceptors, differing from other forms of color blindness, and resulting in central vision loss. Spectral-domain optical coherence tomography (SD-OCT) has been pivotal in evaluating retinal microarchitecture and photoreceptor morphology, showing complete loss of the photoreceptor outer segments, RPE disruption, and outer nuclear layer loss [157]. The functional alterations may precede the structural damage visible in OCT, as at birth, the outer retina can appear intact in OCT even in the presence of non-functional cones [158]. AO is a valuable imaging tool with the capability to detect the morphology of the cone photoreceptors and quantify the cone density. Previous AO studies have provided high-resolution images of individual cone photoreceptors, highlighting a significant decrease in cone density within the fovea [78,159,160,161].

## 5. Congenital Vitreoretinopathies

### Congenital X-Linked Retinoschisis (XLRS)

XLRS (RS1, OMIM #300839: X-linked recessive) is a retinal dystrophy characterized by retinoschisis of the inner and/or neuronal retina, and vision loss in affected men, while carrier females are asymptomatic. This condition is responsible for the majority of cases of congenital retinoschisis: macula (foveal) retinoschisis has been reported in 70–100% of the XLRS cases in younger patients, while in 50% of the cases, peripheral retinoschisis is also observed [162,163,164]. SD-OCT has been pivotal in studying the schisis, its location within the retina, and its extension. OCT studies have shown that the schisis can occur within the inner nuclear layer, outer plexiform layer, outer nuclear layer, ganglion cell layer, and nerve fiber layer, though it is reported to be more prominent in the inner nuclear layer and outer nuclear layer, which is consistent with the retinoschisin on bipolar cells and photoreceptors [165]. More recently, the importance of en face OCT imaging has been reported in phenotyping the different patterns of retinoschisis [166]. Specifically, en face OCT has shown a pattern characterized by multiple hypo-reflective cavitary changes in the parafoveal region at the level of the ganglion cell layer, a spoke-like pattern in the foveal region, a reticular pattern in the parafoveal region within the inner nuclear layer, and multiple separated hypo-reflective polygonal cavities within the outer plexiform layer [166]. One of the limitations of the OCT B-scan cross-sectional imaging is that the retinoschisis within the different retina layers may have similar characteristics, while the en face OCT images may help in providing an understanding the structural alterations specific to each retinal layer, which may ultimately provide insights into the pathophysiology of the schisis [167]. FAF patterns vary from normal FAF findings to decreased autofluorescence within the foveal region, or irregular concentric areas of hyper- and hypo-autofluorescence, or a region of hypo-autofluorescence surrounded by a ring of hyper-autofluorescence [168]. In a phase 1/2 clinical trial assessing gene augmentation therapy in patients with XLRS using a different rAAV (rAAV2tYF-CB-hRS1) vector expressing RS1, cystic cavity volume in OCT imaging was considered as an imaging endpoint. Although this endpoint measure was associated with some changes in the study eyes during the study, the changes were similar to the ones detected in the fellow eye [169]. Structural outcome measures assessed via OCT, such as closure of schitic cavities and changes of clinical retinal architecture, were reported in a phase 1/2a dose-escalation trial of AAV8-RS1 retinal gene transfer [170].

## 6. Limitations, Strengths

Our work has a few limitations. First, the narrative and comprehensive nature of this review does not represent a systematic review of the literature on this topic and does not include any meta-analyses. We limited our description to imaging findings and clinical trial outcome measures across the main IRDs, while we did not associate the different imaging findings with the molecular profile or the electrophysiology, as we focused on the retinal imaging biomarkers. Our manuscript also has some strengths. It represents a wide review of the literature of the main IRDs described in terms of retinal imaging, which plays a pivotal role in the diagnosis, monitoring, and following up of IRDs. As such, we believe it will serve as a useful resource for clinicians evaluating these patients.

## 7. Conclusions

Advances in retinal imaging technology have allowed a more precise characterization of the phenotype of IRDs, and consequently an improvement in the understanding of the pathophysiology of this group of diseases. Over the last decades, a number of new imaging biomarkers in the context of IRDs have been uncovered and employed in clinical trials as repeatable and precise outcome measures and endpoints, facilitating clinical research and therapeutic development for IRDs. Looking to the future, deep-learning approaches that can achieve good performance, even with small training datasets, may aid in uncovering new biomarkers and may automate the process of precisely quantifying biomarkers and endpoints from imaging data. Coupled with further advances in imaging technologies and the broader availability of genetic testing and whole-genome sequencing, we would anticipate further improvements in our ability to correlate phenotype with genotype. Ultimately, we expect these advances to allow us to more precisely diagnose and monitor disease, as well as the response to therapy, which will better care for our patients with IRD.
